# Identification of two *CUL7* variants in two Chinese families with 3‐M syndrome by whole‐exome sequencing

**DOI:** 10.1002/jcla.23265

**Published:** 2020-03-06

**Authors:** Li Hu, Xike Wang, Tingting Jin, Yuanyuan Han, Juan Liu, Minmin Jiang, Shujuan Yan, Xiaoling Fu, Bangquan An, Shengwen Huang

**Affiliations:** ^1^ School of Medicine Guizhou University Guiyang China; ^2^ Prenatal Diagnosis Center Guizhou Provincial People's Hospital Guiyang China; ^3^ Department of Pediatrics Guizhou Provincial People's Hospital Guiyang China; ^4^ Department of Blood Transfusion Guizhou Provincial People's Hospital Guiyang China; ^5^ NHC Key Laboratory of Pulmonary Immunological Diseases Guizhou Provincial People's Hospital Guiyang China

**Keywords:** 3‐M syndrome, CUL7, homozygous variant, short stature, whole‐exome sequencing

## Abstract

**Background:**

3‐M syndrome is a rare autosomal recessive disorder characterized by primordial growth retardation, large head circumference, characteristic facial features, and mild skeletal changes, which is associated with the exclusive variants in three genes, namely *CUL7*, *OBSL1*, and *CCDC8*. Only a few 3‐M syndrome patients have been reported in Chinese population.

**Methods:**

Children with unexplained severe short stature, facial dysmorphism, and normal intelligence in two Chinese families and their relatives were enrolled. Trio‐whole‐exome sequencing (trio‐WES) and pathogenicity prediction analysis were conducted on the recruited patients. A conservative analysis of the mutant amino acid sequences and function prediction analysis of the wild‐type (WT) and mutant CUL7 protein were performed.

**Results:**

We identified a homozygous missense variant (NM_014780.4: c.4898C > T, p.Thr1633Met) in *CUL7* gene in a 6‐month‐old female infant from a non‐consanguineous family, and a homozygous frameshift variant (NM_014780.4: c.3722_3749 dup GGCTGGCACAGCTGCAGCAATGCCTGCA, p. Val1252Glyfs*23) in *CUL7* gene in two affected siblings from a consanguinity family. These two variants may affect the properties and structure of CUL7 protein.

**Conclusion:**

These two rare variants were observed in Chinese population for the first time and have not been reported in the literature. Our findings expand the variant spectrum of 3‐M syndrome in Chinese population and provide valuable insights into the early clinical manifestations and pathogenesis of 3‐M syndrome for pediatricians and endocrinologists.

## 1 INTRODUCTION

3‐M syndrome (MIM #273750, 612921, 614205) is an extremely rare primordial autosomal recessive disorder that is associated with growth retardation. The pathogenesis responsible for this disorder remains largely unknown.^1^, ^2^, ^3^ Patients with 3‐M syndrome often have severe short stature characterized by a poor pre‐ and postnatal growth retardation, large head circumference, characteristic facial features, mild skeletal changes, normal intelligence, and endocrine function. However, these characteristics are not specific and changeless.^4^, ^5^ Characteristic facial features include dolichocephaly, protruding forehead, triangular face, flat nose, long philtrum, full lips, and pointed chin. In some male patients with 3‐M syndrome, hypogonadism and/or hypospadias have occasionally been reported.^3^, ^6^, ^7^, ^8^, ^9^ Apart from intrauterine growth retardation, 3‐M patients usually suffered from feeding difficulties during infancy and postpartum growth retardation. Other clinical characteristics include short neck, square shoulders, slender long tubular bones, tall vertebral bodies, winged scapulae, short thorax, transverse chest groove, pectus carinatum or excavatum, scoliosis, joint hypermobility, clinodactyly, hyperlordosis, developmental dysplasia hip and spina bifida occulta, and prominent heels. These anomalies can be clearly detected by imaging tests.^2^, ^10^

In 1975, 3‐M syndrome was first described by three principal geneticists (Miller, Mukusick, and Malvaux), and the name was subsequently derived from their initials. This disease is occasionally recognized in childhood, in which its prevalence and incidence at birth are low in general populations.^2^, ^9^ To date, fewer than 100 cases of 3‐M syndrome have been reported in the literature. 3‐M syndrome is classified into types I, II, and III, which have been identified to be associated with the exclusive variants in cullin 7 (*CUL7*; MIM *609577), obscurin‐like 1 (*OBSL1*; MIM *610991), and coiled‐coil domain‐containing protein 8 (*CCDC8*; MIM *614145) genes, respectively. However, not all the causative variants in these three genes can account for 3‐M syndrome cases. Among them, *CUL7* appears to be the most predominant pathogenic gene, and the proportion of 3‐M syndrome cases with *CUL7* variants is 77.5%, while *OBSL1* accounts for a relatively small proportion of 16.3%.^3^, ^11^ Most variants in these three genes result in a truncated protein, suggesting that loss‐of‐function variants are responsible for this disease.^12^ It is worth noting that some patients with similar phenotypes do not carry variants in the three genes, indicating that there may be other genes that are responsible for 3‐M syndrome. Given the rarity and heterogeneity of 3‐M syndrome, the patients continue to pose diagnostic and therapeutic challenges.^13^

In this study, by assessing two pediatric families with unexplained short stature children through whole‐exome sequencing (WES) and bioinformatics analysis, we identified two autosomal recessive homozygous variants in *CUL7* gene, including NM_014780.4: c.4898 C > T (p. Thr1633Met) variant and NM_014780.4: c.3722_3749 dup GGCTGGCACAGCTGCAGCAATGCCTGCA (p. Val1252Glyfs*23). These findings can expand the variant spectrum of 3‐M syndrome in Chinese population. Further studies on how these two variants cause 3‐M syndrome will commence in the near future.

## 2 MATERIALS AND METHODS

### 2.1 Subjects

This study was approved by the ethics committee of Guizhou Provincial People's Hospital. Informed written consent was obtained from the parents of the patients.

**Figure 1:**
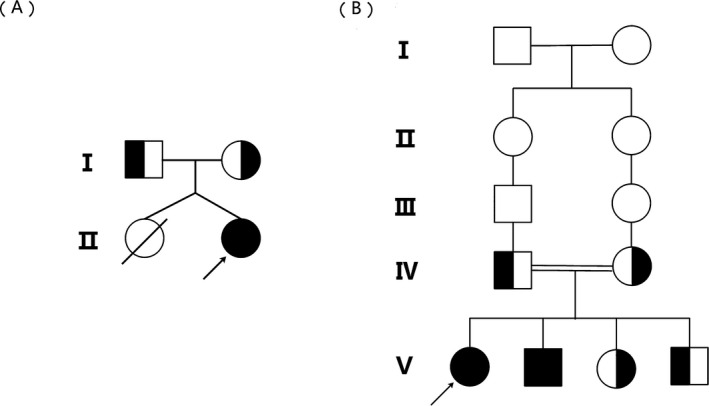
Family pedigree. A, The pedigree of Family A; B, the pedigree of Family B. The arrows indicate the probands

Family A: Patient 1 is a 6‐month‐old female infant who visited the pediatric outpatient department at Guizhou Provincial People's Hospital due to developmental abnormalities. She was the second child in a twin pregnancy. B‐scan ultrasonography indicated the presence of a twin gestation during the fifth month of pregnancy. At the seventh month of pregnancy, a fetus was found dead in the uterus. After half a month, there was no fetal movement, and a cesarean section was performed immediately. Her birth weight was 2700 g. Her parents were of non‐consanguineous marriage, with a smoke‐free wine hobby and no history of exposure to radiation and chemical substances. There were no other affected members in her family. The detailed family pedigree is shown in Figure 1A.

Family B: Two siblings, a 12‐year‐old female child (patient 2) and her 9‐year‐old brother (patient 3), were both born after a normal gestation. They were born to consanguineous parents who are second cousins. The detailed family pedigree is shown in Figure 1B. Although their birth data were not available, their birth length and weight were in normal range based on their mother's review. Growth and developmental delay were noted since their childhood. Considering that the two affected siblings were of short stature, the genetic analysis of 10 exons in growth hormone receptor (*GHR*) gene was initially conducted by Sanger sequencing. However, the results showed that no variant was found in *GHR* gene (data not shown).

### 2.2 Whole‐exome sequencing

Peripheral venous blood samples were collected from all the affected children and their unaffected relatives. Genomic DNA was isolated from peripheral blood leukocytes using a Blood Genomic DNA Kit (Tiangen) according to the manufacturer's instructions. To identify the potential pathogenic variants in the affected patients, trio‐WES was performed on two probands as well as their unaffected parents. The samples were fragmented to 100 ~ 700 bp using an ultrasonic disruptor (Covaris‐S220), followed by DNA library construction using a MyGenostics Standard Library Construction Kit (MyGenostics). The biotin‐labeled whole‐exome probes were hybridized with the constructed DNA libraries, in order to capture and enrich the genes of interest. Next‐generation sequencing was carried out on an Illumina NextSeq 500 Sequencer (Illumina).

### 2.3 Bioinformatics analysis

Bioinformatics analysis was conducted as follows. First, low‐quality and adapter reads were removed from raw data. After quality controlling, sequencing reads were aligned to GRCh37/hg19 using Burrows‐Wheeler alignment (BWA). The SAMtools (http://samtools.sourceforge.net/) was used to identify single nucleotide variants (SNVs) and small indels. Picard MarkDuplicates.jar tool was used to remove or mark the redundant reads during PCR amplification, eliminate variants caused by library amplification, and reduce false positives. Then, SNVs and indels in each sample were recalibrated and called with GATK program. All the identified variants were annotated and classified by ANNOVAR software. The variants with <1% minor allele frequency in all the following databases were excluded: ExAC (http://exac.broadinstitute.org), 1,000 Genomes (http://www.1000genomes.org/), and ESP6500 (http://evs.gs.washington.edu/EVS). Subsequently, the variants were analyzed by independent protein pathogenicity predictors as follows: PolyPhen‐2 (http://genetics.bwh.harvard. edu/pph2/), SIFT (http://sift.jcvi.org/), MutationTaster (http://www.mutationtaster. org/), and CADD (https://cadd.gs.washington.edu/snv). Finally, Online Mendelian Inheritance in Man (OMIM) (https://www.omim.org/), Human Gene Mutation Database (HGMD) (http://www.hgmd.cf.ac.uk/ac/index.php), ClinVar database (https://www.ncbi.nlm.nih.gov/clinvar/), and the American College of Medical Genetics and Genomics (ACMG) and Association for Molecular Pathology (AMP) guidelines were used to classify the pathogenicity of each genetic variation.

### 2.4 Sanger sequencing

Based on the results of WES, the disease‐causing gene variants of each family member and normal control were verified by Sanger sequencing. Primers flanking the regions of exons 20 and 26 in *CUL7* gene were designed by Primer 5.0 and used for PCR amplification. All PCR products were sequenced using an ABI 3130 Genetic Analyzer. Sequence analysis was performed with the Chromas program in DNASTAR analysis package, in order to visually inspect potential base alterations.

### 2.5 Conservative and in silico analysis

We did a conservative analysis of the two mutant amino acid sequences using the Clustal Omega (https://www.ebi.ac.uk/Tools/msa/clustalo/). Further, the three‐dimensional structures of the wild‐type (WT) and mutant CUL7 protein were predicted by using Phyre2 software (http://www.sbg.bio.ic.ac.uk/phyre2/html/page.cgi?id=index); then, Swiss‐PdbViewer 4.04 was conducted to analyze the structural diversity and physical‐chemical changes between WT and mutant CUL7 proteins.

## 3 RESULTS

### 3.1 Clinical evaluation

Physical examination revealed that the length and weight of patient 1 were 62.5 cm (−2 standard deviation [SD]) and 5300 g (−3SD), respectively. Her head circumference was 45 cm (+2SD), while chest circumference was 35 cm. Her specific appearance included slightly prominent forehead, short nose, long philtrum, slightly full lips, short neck, and narrow chest. Unfortunately, there was no figure about her facial appearance, as the permission was not granted by her parents. The muscle tension of the limbs was normal, along with a slight clinodactyly of the fifth finger and prominent heels. Neurological examination revealed no abnormal findings. Admission laboratory testing, such as liver, kidney, and thyroid function tests, was all normal. The computed tomography scan and electroencephalogram examination of the head indicated no abnormalities. Considering the growth delay of the child, growth hormone (GH) stimulation test was performed to rule out the possibility of dwarfism. The levels of GH and insulin‐like growth factor 1 were 15.3 μg/L and 186 ng/L, respectively, while the tandem mass spectrometric analysis showed no abnormality in blood and urine samples.

**Figure 2:**
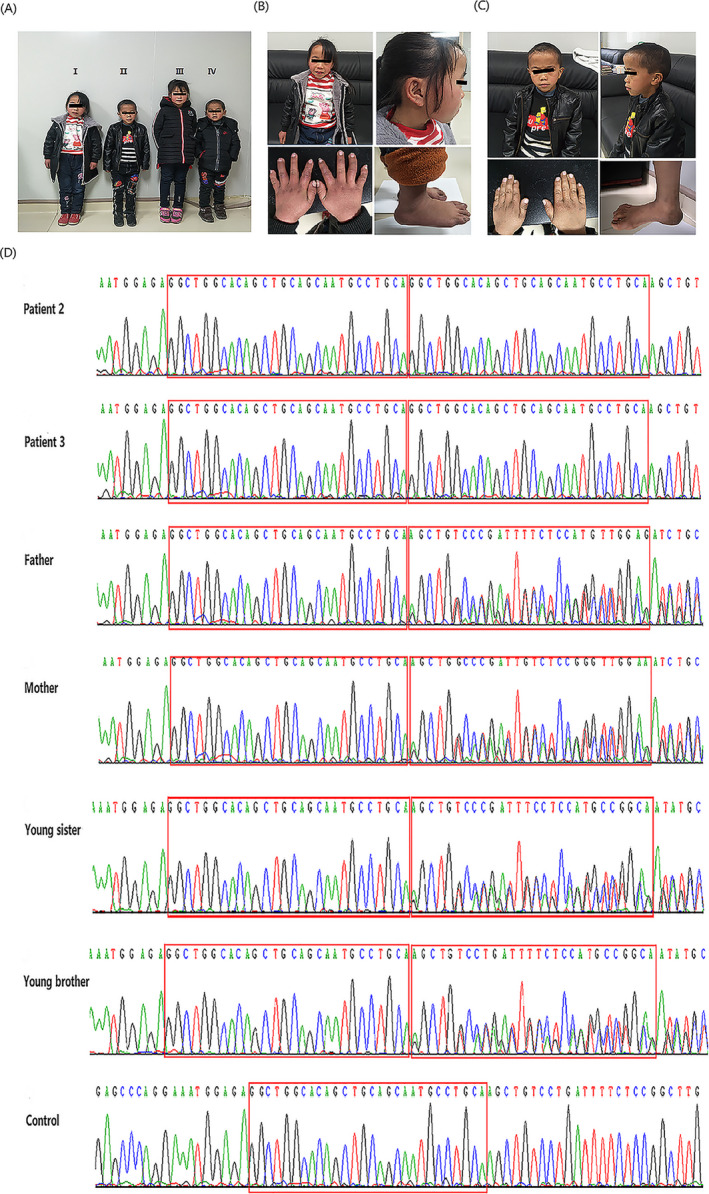
Clinical data and Sanger sequencing of Family B. A, four children of Family B, I is patient 2, II is patient 3, III is young sister, and IV is young brother; B, clinical features of patient 2; C, clinical features of patient 3; D, Sanger sequencing of pathogenic variants in Family B; the red boxes indicate the duplication sequence

Upon physical examination, patients 2 and 3 exhibited severe short stature (102.7 [−9SD] and 101.1 [−6SD] cm, respectively) and poor weight gain (16 500 [−3SD] and 13 200 [−3SD] g, respectively), as shown in Figure 2A. They also displayed numerous physical characteristics, including prominent forehead, fleshy nose tip, long philtrum, prominent mouth, pointed chin, clinodactyly of the fifth finger, and prominent heels. The brother displayed a typical face evidently, such as easily recognized triangular face (Figure 2B,C). Through a skeletal survey, the bone age of the two affected siblings delayed slightly. They underwent some examinations for short stature, including routine biochemical tests; liver, kidney, and thyroid function tests; GH and GH stimulation tests; and abdominal ultrasound scanning. All parameters were within normal range. No skin lesion or hepatosplenomegaly was observed. There are another two young siblings in this family: a 7‐year‐old female and a 5‐year‐old male whose phenotypes were similar to their unaffected parents, with the height of 119.2 (−2SD) and 106.2 (−2SD) cm as well as the weight of 23 000 (−1SD) and 18 000 (−2SD) g, respectively (Figure 2A).

### 3.2 Results of genetic analysis

**Figure 3:**
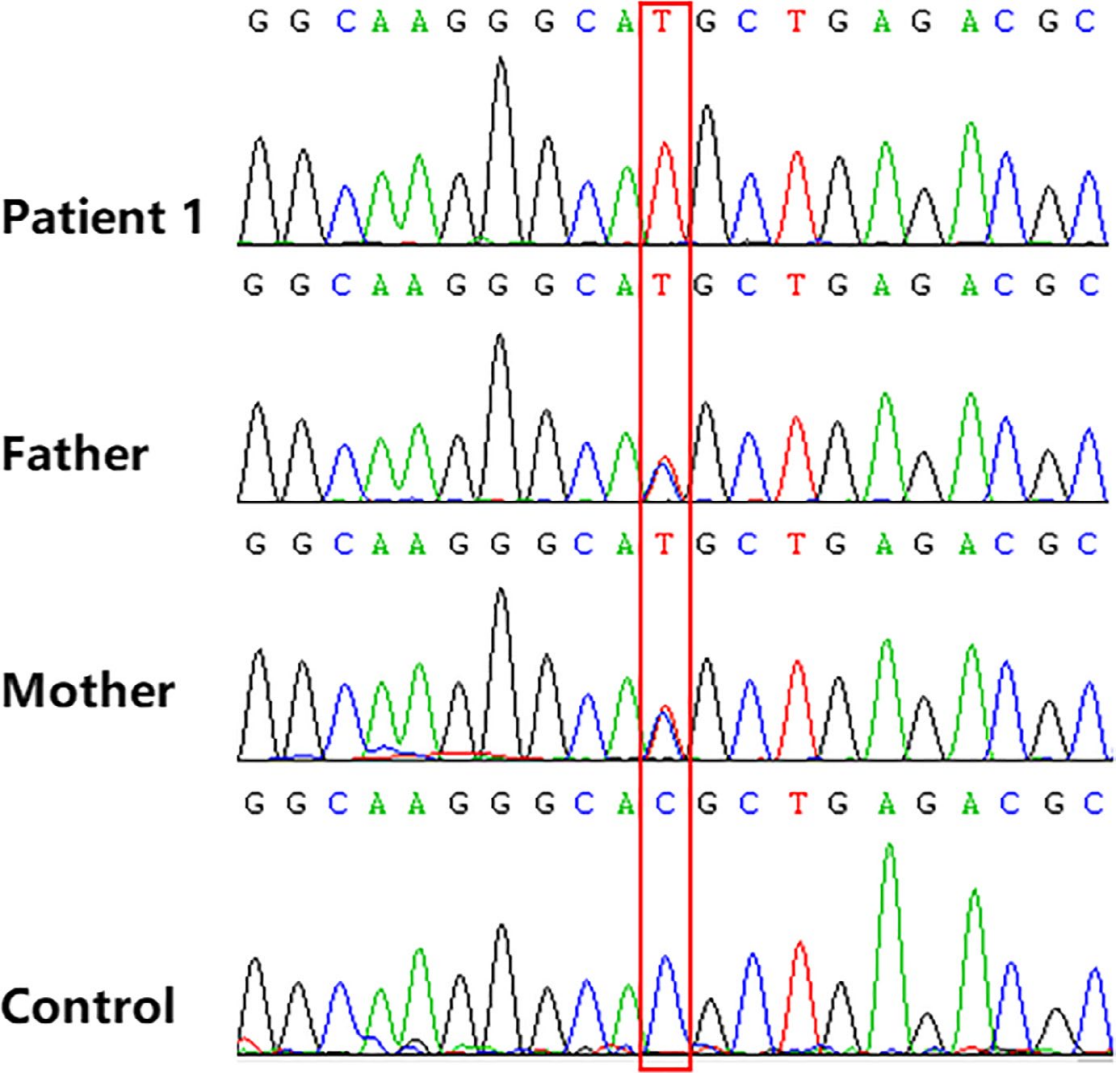
Sanger sequencing of Family A; the red box indicates the mutation

In patient 1 from Family A, a homozygous missense variant, namely NM_014780.4: c.4898 C > T (p. Thr1633Met), was detected in the exon 26 of *CUL7* gene. As shown in Figure 3, her parents were heterozygous carriers of the c.4898C > T variant. Moreover, the allele frequencies of c.4898 C > T in the East Asia population were 0.026303, 0.0337, and 0.0279099 in ExAC, 1000 Genomes, and gnomAD database, respectively. The results of PolyPhen‐2 estimated that this variant was probably damaging; those of SIFT indicated that it was deleterious, with a tolerance score of 0.21; and those of MutationTaster revealed that it was a polymorphic variant. The CADD score of this variant was 29.3, indicating that this variant is deleterious. According to the ACMG/AMP standards and guidelines, the classification of this missense variant was VUS (variant of uncertain significance) and the criterion was BS1.

In patient 2 from Family B, a homozygous duplication variant of 28‐bp GGCTGGCACAGCTGCAGCAATGCCTGCA (NM_014780.4: c.3722_3749 dup 28) was detected in the exon 20 of *CUL7* gene. Bioinformatics analysis predicted a frameshift variant and a preterminal stop codon (p. Val1252Glyfs*23) within the duplication, which could lead to a truncated cullin 7 protein. The allele frequency of this variant in the East Asia population was 0.000119 in ExAC database. Based on Sanger sequencing data, her affected brother (patient 3) carried the same variant, while their unaffected parents and unaffected two younger siblings were all in a heterozygous state (Figure 2D). According to the ACMG/AMP standards and guidelines, the classification of this variant was LP (likely pathogenic) and the criteria were PVS1 and PM2. Therefore, the homozygous 28‐bp duplication is most likely to be associated with type I 3‐M syndrome.

### 3.3 Results of conservative and in silico analysis

**Figure 4:**
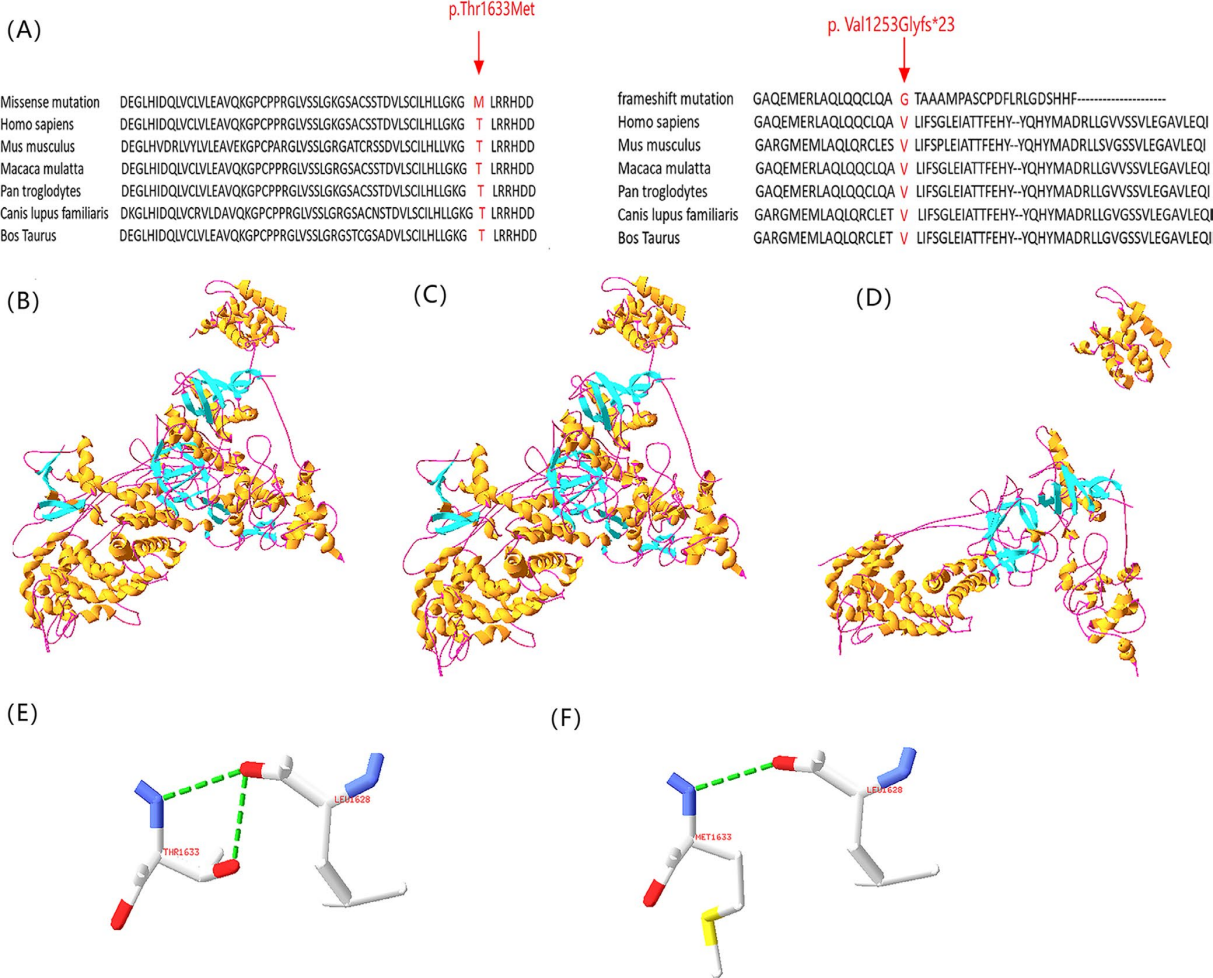
Conservative and in silico analysis. A, Protein sites with amino acid change are evolutionarily conserved among six species. Protein locus with mutations is highlighted with black arrows; B, the structure prediction of wild‐type CUL7 protein; C, the structure prediction of missense mutation CUL7 protein; D, the structure prediction of frameshift mutation CUL7 protein; E, the partial enlargement of missense mutation CUL7 protein; F, the partial enlargement of frameshift mutation CUL7 protein

Both the loci of the missense variant (c.4898 C > T, p. Thr1633Met) and frameshift variant (c.3722_3749 dup 28, p. Val1252Glyfs*23) were most evolutionarily conserved in six species (Figure 4A), suggesting these two variants were likely pathological. In the WT protein, the Thr at position 1633 forms two H‐bonds with Leu at position 1628 (Figure 4E). When Met replaced Thr, although the complex structure did not change (Figure 4B), one H‐bonds break and disappeared. Besides, the side chain of amino acid residues also changed (Figure 4F). Obviously, the frameshift variant resulted in a truncated protein compared with the WT one (Figure 4D).

## 4 DISCUSSION

3‐M syndrome is an under‐diagnosed disorder characterized by primordial growth retardation, large head circumference, characteristic facial features, and mild skeletal changes. Given that 3‐M syndrome is an autosomal recessive disorder, early diagnosis and genetic counseling are particularly important for the patients and their relatives.^11^ However, pediatricians tend to ignore the diagnosis and genetic analysis of 3‐M syndrome patients with dysmorphic features, which can result in a series of unnecessary examination costs and inappropriate treatment. For an accurate diagnosis of 3‐M syndrome, apart from its clinical features, imaging analysis of the spine and long bones as well as genetic analysis of disease‐causing genes may be particularly helpful. Besides, GH has been found to be a useful treatment for 3‐M syndrome in few studies,^14^, ^15^ while other findings demonstrated that such treatment exerts no beneficial effect.^5^, ^16^

3‐M syndrome has been causally related to *CUL7*, *OBSL1,* and *CCDC8* variants, but the underlying mechanisms remain largely unknown.^1^, ^6^, ^17^ Huber et al reported for the first time that *CUL7* variants are convincingly associated with 3‐M syndrome in 2005. Subsequently, *CUL7* variants have been identified from homozygosity mapping in consanguineous families, revealing a locus gene on chromosome 6p21.1.^6^ To date, there were 70 *CUL7* variants recorded in HGMD database.^18^ A high proportion of variants has been found throughout the entire *CUL7* gene, and more than half of the variants are located in the cullin domain. The majority of *CUL7* variants can result in a premature stop codon, leading to a complete loss of CUL7 protein. However, a small percentage of missense variants is predicted to affect the production of non‐functional protein products.^2^

The CUL7 protein is the seventh member of the cullin family, which contains an evolutionarily conserved cullin domain and a DOC domain. It can function as a crucial scaffold protein forming component of an E3 ubiquitin ligase complex that promotes cytoplasmic protein degradation by interacting with a heterodimer (composed of Skp1 adapter protein binding to Fbw8 protein) and ROC1 RING finger protein.^3^, ^6^, ^8^, ^12^, ^19^ A previous study has shown that CUL7 can promote cell growth by partially antagonizing p53 through biochemical and functional analyses.^20^ When fertile p185+/− mice intercross each other, p185−/− mice with perinatal lethality, intrauterine growth restriction, and placental defects, which indicated that CUL7 plays a critical role in developmental processes.^21^

There are only 10 cases of 3‐M syndrome that have been reported in Chinese population, including six cases reported in 2017,^18^ and the other four cases were reported in Chinese journals.^22^, ^23^ To the best of our knowledge, the missense variant and frameshift variant in our study were observed in Chinese population for the first time and recorded in ClinVar and ExAC databases, respectively. The clinical phenotypes, case characteristics, and functional analysis of these two variants have not been reported in the literature, which could be attributed to the rarity of 3‐M syndrome. Together with our findings, 13 cases were reported in Chinese population at present, most of which were caused by the *CUL7* gene variants.

The missense *CUL7* variant c.4898 C > T occurred in cullin_Nedd8 domain that is the neddylation site of cullin proteins which are a family of structurally related proteins containing an evolutionarily conserved cullin domain. Neddylation is a remarkably intricate biochemical process that plays an indispensable role in the regulation of cell cycle and embryogenesis.^24^ The disappearance of an H‐bond and changes in the side chain of amino acid residue are caused by the variant, which could lead to conformational instability of CUL7 protein and change its activities. In addition, patient 1 was treated with GH for 6 months, but failed to exhibit the desired effect, which is consistent with previous findings.^16^ It is possible that the variants in *CUL7* gene may strongly influence the effect of GH on longitudinal growth.

As a consequence of consanguineous marriage (second cousins), two affected children in family B with *CUL7* homozygous frameshift variant that may be inherited from their great‐great‐grandmother or great‐great‐grandfather. It is absent in the gnomAD and ClinVar database, but in ExAC with frequency 0.000119. Therefore, such low‐frequency variant meets the classification of likely pathogenic. This variant occurred in the cullin domain and led to an obvious truncated protein. Cullins are a family of hydrophobic proteins that act as scaffolds for ubiquitin ligases (E3). Cullin‐RING ubiquitin ligases (CRLs) play an essential role in targeting proteins for ubiquitin‐mediated destruction.^25^

Given the above, these two variants may affect the properties and structure of the protein. Therefore, these two variants most possibility to be the pathogenesis of short stature in affected children. Together with clinical features and genetic analysis, these three affected children in the two families were ultimately diagnosed as suffering from 3‐M syndrome.

However, one of the limitations of this study is that we have not done functional experiments for these two variants. Besides, although we followed up patient 1 with six months of GH therapy and found that it was ineffective, it is a pity that we have not followed up observation of the efficacy on the three children for long‐term treatment. Further studies are necessary to determine how the two variants contribute to short stature.

Herein, we demonstrated that WES could serve as a useful tool for undiagnosed patients with 3‐M syndrome. Our findings expand the variant spectrum of 3‐M syndrome in Chinese population and provide valuable insights into the early clinical manifestations and pathogenesis of 3‐M syndrome for pediatricians and endocrinologists. Further studies are ongoing and will hopefully shed light on the pathogenic function of *CUL7* in 3‐M syndrome.

## AUTHOR CONTRIBUTIONS

LH, XW, XF, and SH designed the study and collected the samples; LH, TJ, YH, and JL performed experimental work, analyzed the data, and drafted the manuscript. SY, MJ, XF, BA, and SH drafted the final version of the manuscript. All authors read and approved the final manuscript.
